# The Content of Dietary Melatonin in 119 Food Items and Its Relationship With Chronic Diseases: Results of the CUME+ Study

**DOI:** 10.1111/jhn.70193

**Published:** 2026-01-26

**Authors:** Gilmara Alves Zanirate, Josefina Bressan, Arieta Carla Gualandi Leal, Adriano Marçal Pimenta, Helen Hermana Miranda Hermsdorff

**Affiliations:** ^1^ Department of Nutrition and Health, Laboratory of Clinical Analysis and Genomics Laboratory of Energy Metabolism and Body Composition Viçosa Minas Gerais Brazil; ^2^ Departament of Nursing Universidade Federal do Paraná Curitiba Paraná Brazil

**Keywords:** cohort study, depression, food intake, obesity

## Abstract

**Background:**

Dietary melatonin, naturally occurring in plant‐ and animal‐based foods, has been linked to beneficial effects on sleep, mood and metabolic health. Although evidence suggests that food‐derived melatonin may elevate circulating levels, few studies have assessed its intake through habitual diets or explored associations with chronic disease outcomes in adults.

**Methods:**

We conducted a cross‐sectional analysis within an open cohort of university graduates (baseline data). Dietary intake was evaluated using a validated 144‐item Food Frequency Questionnaire (FFQ), and melatonin intake was estimated based on published concentrations. Sleep duration was also assessed through the questionnaire. Associations between dietary melatonin and selected health outcomes, including depression, obesity, hypertension, type 2 diabetes mellitus, metabolic syndrome, dyslipidemia, obstructive sleep apnoea and sleep duration, were examined across quintiles of intake using logistic regression and Poisson regression models with robust variance, adjusted for potential confounders.

**Results:**

Melatonin content was assigned to 82.6% of the FFQ items, with concentrations ranging from 0 to 169.9 ng/g. Among 8320 participants, most were women (67.7%), with a mean age of 35.9 ± 9.6 years; 48.1% were single. Mean dietary melatonin intake was 25554.7 ± 13876.2 ng/day. In multivariate models, obesity was inversely associated with melatonin intake in Q2 (IRR 0.81, 95%CI 0.69–0.96); Q3 (IRR 0.72, 95%CI 0.60–0.86) and Q4 (IRR 0.79, 95%CI 0.67–0.94). Depression was inversely associated in Q3 (IRR 0.79, 95%CI 0.67–0.93) and Q4 (IRR 0.79, 95%CI 0.67–0.94), suggesting a nonlinear dose–response pattern.

**Conclusion:**

Dietary melatonin intake was inversely associated with obesity and depression, with a non‐linear dose–response pattern observed for depression. No significant associations were found with other chronic conditions or sleep duration. Longitudinal and experimental studies are needed to confirm these findings and clarify underlying mechanisms.

## Summary

- Melatonin content estimated in 119 food items from validated FFQ.
- Mean dietary intake of melatonin reaches ~25500 ng/day among 8320 adults.
- Coffee, beans/lentils and rice are the main contributors to the melatonin intake.
- Higher melatonin intake is inversely associated with obesity risk.
- There is a nonlinear inverse association between dietary melatonin and depression.
- Dietary melatonin no present consistent associations with hypertension, diabetes or sleep duration.

## 1 Introduction

Dietary melatonin refers to its natural presence in plant‐ and animal‐derived foods [1, 2]. First identified in 1995 [3, 4], it has since been detected in meats, fish, eggs, cereals, fruits, milk, coffee, wine and beer [2, 5, 6]. However, no consolidated database currently reports melatonin concentrations across food items.

Although levels in foods are lower than in supplements, evidence suggests that melatonin‐rich diets can elevate circulating levels [6, 7, 8]. Compared with pharmacological supplementation, increasing melatonin intake through dietary sources may provide physiological doses that align more closely with endogenous rhythms and avoid suprapharmacological exposure [9, 10]. Given the global burden of sleep disorders, which affect up to one‐third of adults worldwide [11], depression, which impacts 5.7% of adults globally, over 280 million people [12], and obesity, with 890 million adults living with obesity in 2022 [13], dietary melatonin could represent a complementary strategy to mitigate these conditions. Evidence of protective associations with metabolic, neurobehavioral and inflammatory outcomes further supports the relevance of examining habitual dietary intake [8, 14, 15]. Thus, dietary melatonin may represent a feasible public health strategy to complement existing approaches for the prevention of chronic conditions.

Once absorbed, melatonin crosses the blood–brain barrier and acts on various tissues, including the brain [16, 17]. Accordingly, consumption of melatonin‐rich foods has been linked to improvements in sleep duration [18, 19, 20, 21, 22], sleep quality [23, 24, 25, 26], depressive symptoms [18, 23, 27, 28] and adiposity [27, 28].

Beyond circadian regulation, melatonin exhibits antioxidant, anti‐inflammatory, immunomodulatory and metabolic effects [8, 9, 29], which may underlie its potential health benefits. Observational studies suggest inverse associations with all‐cause mortality [14] and liver cancer incidence [15]. Nonetheless, few investigations have assessed dietary melatonin intake in habitual diets or its relationship with chronic conditions in adults.

This study aimed to compile scientific data on the melatonin content of food items included in the Food Frequency Questionnaire (FFQ) used in an open cohort of university graduates, and to examine associations between dietary melatonin intake and selected health outcomes in adults.

## 2 Subjects and Methods

### 2.1 Study Design

The CUME+ study is an open, prospective cohort of graduates [30]. It investigates the impact of food groups, nutrients, dietary patterns and nutritional transition on noncommunicable diseases (NCDs), including obesity, diabetes, hypertension, dyslipidemia, cardiovascular disease and cancer [30]. In this context, ‘nutritional transition’ refers to the shift in dietary patterns characterised by increased consumption of processed and ultra‐processed products and reduced intake of traditional staple foods, a phenomenon widely recognised as a driver of rising rates of chronic diseases [31, 32]. Design and recruitment details are described elsewhere [30]. The study was approved by the Human Research Ethics Committees of the Federal University of Viçosa (approval number 6.048.479/2023; CAAE 67808923.7.1001.5153). All participants read the informed consent form and provided their agreement online before completing the questionnaire (https://www.projetocume.com.br/questionario).

### 2.2 Study Population

In the CUME+ study, 9409 participants completed the baseline questionnaire in 2016, 2018, 2020, 2022 or 2024. Participants were excluded if they were not residing in the target country (*n* = 429), pregnant women (*n* = 335), and those with implausible energy intake (< 500 or > 6000 kcal/day; *n* = 325) [33]. The final sample comprised 8320 adults (Supporting Material 1).

### 2.3 Data Collection

For data collection, we used a self‐administered baseline online questionnaire (Q_0), which was divided into two parts and accessed at http://www.projetocume.com/questionario.

The first included 83 questions on sociodemographics, lifestyle, morbidity, medication use, clinical history and anthropometry. The second comprised a validated 144‐item FFQ [34], plus questions on supplements, cooking practices and dietary habits.

### 2.4 Outcomes Variables

#### 2.4.1 Outcomes Included

a. **Obesity**: Body mass index (BMI) ≥ 30 kg/m², based on validated self‐reported data [35], and classified according to World Health Organization criteria [36];
b. **Obstructive sleep apnoea** (OSA): self‐reported diagnosis;
c. **Depression**: self‐reported medical diagnosis; sedative/antidepressant use without diagnosis was not classified as a case. Validation showed 81% agreement (kappa = 0.62) [37];
d. **Hypertension**: Systolic blood pressure (SBP) ≥ 140 mmHg, diastolic blood pressure (DBP) ≥ 90 mmHg, medication use or physician diagnosis [35, 38];
e. **Type 2 diabetes mellitus** (T2DM): self‐reported diagnosis, fasting glucose > 126 mg/dL, use of antidiabetics or physician diagnosis during follow‐up [35, 39];
f. **Metabolic syndrome**: Defined by International Diabetes Federation (IDF) criteria [40], requiring central obesity (BMI ≥ 30 kg/m²) plus plus two or more of the following: Triglycerides ≥ 150 mg/dL or treatment for hypertriglyceridemia; high‐density lipoprotein cholesterol (HDL‐c) < 40 mg/dL (men) or < 50 mg/dL (women), or treatment for low HDL‐c; SBP ≥ 130 mmHg or DBP ≥ 85 mmHg, or treatment for hypertension; Fasting glucose ≥ 100 mg/dL or diagnosis of T2DM [35];
g. **Dyslipidemia**: ≥ 1 lipid abnormality (triglycerides ≥ 150 mg/dL, total cholesterol ≥ 200 mg/dL, low‐density lipoprotein cholesterol (LDL‐c) ≥ 130 mg/dL, HDL‐c < 40 mg/dL for men/< 50 mg/dL for women) [35, 41].
h. **Sleep duration:** Defined as short sleep duration (< 7 h/day) or normal sleep duration (≥ 7 h/day) according to the National Sleep Foundation's criteria [42].

### 2.5 Food Intake

Dietary intake was assessed using a validated FFQ, which had been previously tested for reproducibility and validity in this cohort, with precautions to account for seasonal food consumption (24 h collected in two different seasons) [34]. Frequencies were converted to daily intake (g/mL). Nutrient intake was calculated using national [43, 44] and international food composition tables [45], and adjusted for energy intake using the residual method [46].

### 2.6 Exposure Variable

Dietary melatonin content was estimated from literature and standardised to ng/g. When multiple values existed, the mean was used. In absence of data, values from similar foods or preparation forms were applied. Foods without estimable values were excluded. For preparations, melatonin was calculated from ingredients. For foods with melatonin concentration reported only in dry matter, those values were used. When values were available for both dry and fresh matter, only the fresh matter values were considered when calculating the mean, to ensure consistency across the dataset, regardless of the analytical method used. Individual intake was computed by multiplying melatonin content by daily food consumption and adjusted for energy [46].

### 2.7 Covariates

Covariates included age (categorised), sex, family income (< 5, 5–10, ≥ 10 minimum wage [MW]), binge drinking (≥ 4 drinks for women, ≥ 5 for men, in a single occasion during the last 30 days), smoking status (never, former or current smoker), screen time (hours/day), physical activity (≥ 150 min/week moderate or ≥ 75 min/week vigorous) [47], sleep duration (< 7 or ≥ 7 h/day) [42] and medication use (sedatives, antidepressants, weight‐control drugs).

### 2.8 Statistical Analysis

Analyses were performed in Stata 13.1 (https://www.stata.com), with 5% significance. Categorical variables were described as frequencies; quantitative variables as medians and interquartile ranges. Pearson's chi‐squared and Kruskal–Wallis tests were used. Melatonin intake was categorised into quintiles, and all regression models used energy‐adjusted melatonin intake to control for total caloric intake. Poisson regression with robust variance was applied for outcomes with prevalence > 10%; logistic regression for those < 10%, following best practices [48, 49, 50]. Results were reported as ORs or IRRs with 95% confidence intervals. Confounders were identified via directed acyclic graph (Supporting Material 2).

## 3 Results

Among 8320 participants, most were women (67.7%), with a mean age of 35.9 ± 9.6 years; 48.1% were single. Regarding lifestyle, 33.3% reported sleeping less than the recommended duration (< 7 h/day), 73.6% consumed alcohol, and 78.6% had never smoked. Additional characteristics are presented in Table 1. The most prevalent health conditions were dyslipidemia (33.5%), depression (13.8%), obesity (13.2%) and hypertension (11.8%) (Supporting Material 3).

**Table 1:** Sociodemographic and lifestyle characteristics across dietary melatonin quintiles in CUME+ study participants (2016–2024).

Variables	Total sample *n* = 8320	Dietary melatonin, ng/day					*p* value
		Q1 (< 14965.4) *n* = 1664	Q2 (14965.4–20567.7) *n* = 1664	Q3 (20567.7–26051.8) *n* = 1664	Q4 (26051.8–34,426.5) *n* = 1664	Q5 (≥ 34426.5) *n* = 1664	
**Sociodemographic variables**							
**Sex, *n* (%)**							
Female	5635 (67.7)	1238 (74.4)	1236 (74.3)	1187 (71.3)	1112 (66.8)	862 (51.8)	**< 0.001**
Male	2685 (32.3)	426 (25.6)	428 (25.7)	477 (28.7)	552 (33.2)	802 (48.2)	
**Age (years)**							
18–29	2466 (29.7)	593 (35.7)	539 (32.4)	445 (26.7)	450 (27.1)	439 (26.4)	**< 0.001**
30–39	3445 (41.4)	669 (40.2)	677 (40.7)	708 (42.6)	698 (42.0)	693 (41.7)	
40–49	1518 (18.2)	244 (14.7)	261 (15.7)	316 (19.0)	328 (19.7)	369 (22.2)	
50–59	689 (8.3)	119 (7.1)	139 (8.3)	144 (8.6)	157 (9.4)	130 (7.8)	
≥ 60	199 (2.4)	38 (2.3)	48 (2.9)	51 (3.1)	30 (1.8)	32 (1.9)	
**Marital status, *n* (%)**							
Single	3999 (48.1)	879 (52.8)	836 (50.2)	760 (45.7)	763 (45.9)	761 (45.7)	**< 0.001**
Married	3801 (45.7)	694 (41.7)	745 (44.8)	785 (47.1)	797 (47.9)	780 (46.9)	
Divorced	401 (4.8)	80 (4.8)	60 (3.6)	91 (5.5)	79 (4.7)	91 (5.5)	
Widowed/others	119 (1.4)	11 (0.7)	23 (1.4)	28 (1.7)	25 (1.5)	32 (1.9)	
**Household income, *n* (%)**							
< 5 MW	6153 (73.9)	1208 (72.6)	1204 (72.4)	1206 (72.5)	1253 (75.3)	1282 (77.0)	**0.016**
5–10 MW	1676 (20.2)	342 (20.6)	350 (21.0)	357 (21.4)	323 (19.4)	304 (18.3)	
≥ 10 MW	491 (5.9)	114 (6.8)	110 (6.6)	101 (6.1)	88 (5.3)	78 (4.7)	
**Occupational status, *n* (%)**							
Retired/homemaker	196 (2.4)	46 (2.7)	39 (2.3)	44 (2.6)	33 (2.0)	34 (2.0)	0.663
Unemployed	589 (7.1)	121 (7.3)	129 (7.8)	107 (6.5)	114 (6.8)	118 (7.1)	
Student	1377 (16.5)	291 (17.5)	258 (15.5)	265 (15.9)	276 (16.6)	287 (17.3)	
Employed	6158 (74.0)	1206 (72.5)	1238 (74.4)	1248 (75.0)	1241 (74.6)	1225 (73.6)	
**Health behaviours**							
**Smoking status, *n* (%)**							
Never smoked	6544 (78.6)	1378 (82.9)	1369 (82.3)	1335 (80.2)	1283 (77.1)	1179 (70.8)	**< 0.001**
Former smoker	1005(12.1)	152 (9.1)	185 (11.1)	191 (11.5)	215 (12.9)	262 (15.8)	
Current smoker	771 (9.3)	134 (8.0)	110 (6.6)	138 (8.3)	166 (10.0)	223 (13.4)	
**Binge drinking, *n* (%)**							
No	5025 (60.4)	1084 (65.1)	1021 (60.4)	1005 (60.4)	969 (58.2)	946 (56.8)	**< 0.001**
Yes	3295 (39.6)	580 (34.9)	643 (38.6)	659 (39.6)	695 (41.8)	718 (43.2)	
**Sleep duration, *n* (%)**							
Recommended (≥ 7 h)	5554 (66.7)	1113 (66.9)	1125 (67.6)	1134 (68.2)	1135 (68.2)	1047 (62.9)	**0.006**
Short sleep (< 7 h)	2766 (33.3)	551 (33.1)	539 (32.4)	530 (31.8)	529 (31.8)	617 (37.1)	
**Physical activity level, *n* (%)**							
Active	4714 (56.7)	945 (56.8)	981 (58.9)	949 (57.0)	910 (54.7)	929 (55.8)	0.112
Moderately active	1681 (20.2)	339 (20.4)	320 (19.3)	347 (20.8)	361 (21.7)	314 (18.9)	
Inactive	1925 (23.1)	380 (22.8)	363 (21.8)	368 (22.2)	393 (23.6)	421 (25.3)	
**Food intake**							
Energy, kcal/day	2194.7 (1683.8–2867.9)	2536.5 (1926.8–3474.1)^a^	2040.1 (1536.5–2610.7)^b^	1,990.7 (1547.8–2565.4)^b^	2117.7 (1657.3–2691.9)^c^	2397.7 (1850.6–3063.0)^d^	**< 0.001**
% CHO	43.9 (37.9–49.9)	40.2 (33.6–46.3)^a^	43.3 (37.8–49.1)^b^	44.4 (38.4–50.3)^c^	45.0 (39.9–51.0)^d^	46.2 (40.3–52.0)^e^	**< 0.001**
% Protein	17.4 (14.9–20.3)	18.2 (15.2–21.9)^a^	17.6 (15.2–20.3)^b^	17.3 (14.8–20.4)^c^	17.2 (14.8–19.8)^c^	16.9 (14.5–19.5)^d^	**< 0.001**
% Fat	36.3 (31.7–41.0)	39.7 (35.0–44.3)^a^	37.8 (32.4–41.5)^b^	35.9 (31.5–40.4)^c^	35.0 (30.9–39.1)^d^	34.5 (29.9–38.9)^e^	**< 0.001**
Cholesterol, mg/day	355.4 (282.9–448.5)	393.1 (295.0–522.0)^a^	365.1 (301.9–448.8)^b^	361.1 (297.5–444.3)^b^	346.6 (283.7–425.3)^c^	316.7 (240.0–405.4)^d^	**< 0.001**
MUFA, g/day	35.7 (31.0–40.8)	37.9 (32.4–44.7)^a^	36.9 (32.6–41.2)^b^	35.7 (31.3–40.0)^c^	35.2 (30.9–39.8)^d^	33.2 (27.6–38.2)^e^	**< 0.001**
PUFA, g/day	19.0 (16.1–22.4)	19.1 (15.2–23.5)	19.0 (16.3–22.1)	18.7 (16.1–21.9)	19.0 (16.2–22.2)	19.1 (16.1–22.8)	0.156
SFA, g/day	34.5 (29.4–39.5)	36.0 (30.5–42.2)^a^	35.0 (30.3–39.5)^b^	34.2 (29.9–38.9)^c^	33.9 (29.3–38.3)^d^	32.4 (27.3–38.4)^e^	**< 0.001**
Dietary fibre, g/day	25.4 (20.6–31.4)	23.1 (17.5–29.9)^a^	24.3 (20.9–30.4)^b^	26.1 (21.6–31.6)^c^	25.9 (21.6–31.5)^c^	26.5 (21.2–33.8)^d^	**< 0.001**

*Note:* Statistical tests: chi‐square (*n*, %) and Kruskal‐Wallis (interquartile range, P25–P75). Means sharing the same letter do not differ significantly (Dunn's test, 5% level). Binge drinking: ≥ 4 drinks (women) or ≥ 5 drinks (men) in a single occasion during the last 30 days. Bold *p* values are Statistically significant.

Abbreviations: %CHO, carbohydrate (% of total energy); MUFA, monounsaturated fatty acids; MW, minimum wage (R$880 in 2016; R$954 in 2018; R$1,045 in 2020; R$1,212 in 2022; R$1,518 in 2024); PUFA, polyunsaturated fatty acids; SFA, saturated fatty acids.

Of the 144 food items listed in the FFQ, melatonin concentrations were assigned to 119 (82.6%) based on a literature review (Table 2). Values ranged from 0 ng/g (potatoes) to 169.9 ng/g (breakfast cereal). Full coverage (100%) was achieved for dairy, cereals/legumes and vegetables; coverage ranged from 95.6% for meat/fish to 56% for processed and ultra‐processed foods.

**Table 2:** Melatonin content of the foods (*n* = 119) from the FFQ of the CUME+ study (2016–2024).

Food group		Melatonin (ng/g ou ng/mL)	Min–max values (ng/g ou ng/mL)		Ref.	Analytical method	
Dairy							
Whole milk		0.015	0.004–0.039		[51]	ELISA	
Skim milk^a^		0.015			[52]	RIA	
Semi‐skimmed milk^a^		0.015			[53]	LC‐MS/MS	
					[54]	EIA	
					[55, 56]	ELISA	
					[26]	RIA	
Soy milk powder		9.28			[57]	HPLC‐FD	
Whole yogurt		0.127			[58]	LC‐MS/MS	
Skim/light yogurt^b^		0.127					
Cream cheese^a^		0.015					
Light cream cheese^a^		0.015					
Cheese (mozzarella, provolone, minas, canastra and prato)^b^		0.127					
Cottage cheese^b^		0.127					
Ricotta cheese^b^		0.127					
Meats and fish							
Mortadella, salami and fatty ham^c^		1.54					
Turkey breast and Chester^d^		1.61					
Beef (steak)		2.1			[59]	HPLC	
Beef (cubes and pieces)		2.1					
Chicken with skin		2.3			[59]	HPLC	
Skinless chicken		2.3					
Pork		2.5			[59]	HPLC	
Lamb and goat meat		1.6			[59]	HPLC	
Soy meat and tofu^e^		3.120			[60]	HPLC	
Sun‐dried beef (jerked beef)^f^		2.1					
Smoked meats^g^		2.5					
Offal (heart, liver and gizzard)		1.1			[59]	HPLC	
Sausage^h^		1.2					
Large sausage and linguiça^h^		1.2					
Boiled chicken egg		6.1			[59]	HPLC	
Bacon and pork fat^g^		2.5					
Meatballs		3.18					
Suchi and sachimi		2.42					
Sardines and tuna (canned)^i^		3.7					
Shrimp and shellfish		—					
Salmon		3.7			[59]	HPLC	
Codfish^i^		3.7					
Other fish^i^		3.7					
Cereals and Legumes							
French bread		0.342	0.138–0.480		[58]	LC‐MS/MS	
					[61]	LC–ESI‐MS/MS	
Sliced bread^j^		0.342					
Toast^j^		0.342					
Whole grain bread (rye, wheat and oats)		0.342					
Light bread^j^		0.342					
Sweet bread^j^		0.342					
Cheese bread^u^		0.977					
Breakfast cereal^u^		169.936					
Oats, wheat germ and granola^u^		60.94					
Cereal bar		60.94					
Rice		89.356	11.0–264.0		[62]	HPLC	
					[63]	HPLC‐FD	
Brown rice		42.95			[63]	HPLC‐FD	
Pasta^u^		3.17					
Lasagna, cannelloni and rondelli^u^		2.84					
Gnocchi^u^		3.17					
Polenta and cornmeal mush^u^		42.48					
Fried polenta		42.48					
Canjiquinha (cracked corn porridge)^u^		22.53					
Pizza^u^		2.84					
Cassava flour and breadcrumbs^j^		0.342					
Corn flour^k^		1.415					
Beans and lentils^l^		54.79			[57]	HPLC‐FD	
Chickpeas^l^		54.79					
Fats and oils							
Butter		—					
Margarine		—					
Mayonnaise^u^		1.782					
Light margarine and light mayonnaise^u^		0.093					
Olive oil		0.087	0.03‐0.119		[64]	LC–MS/MS	
					[65]	HPLC‐FD	
Canola oil^m^		0.97					
Sunflower oil		0.053	0.050–0.055		[64]	LC–MS/MS	
					[65]	HPLC‐FD	
Corn oil^m^		0.97					
Soybean oil		0.19			[65]	HPLC‐FD	
Pork fat and lard		*—*					
Fruits							
Avocado		—					
Pineapple		0.311	0.278–0.360		[66]	RIA	
					[4]	RIA	
					[67]	HPLC‐FD, confirmed by ELISA	
					[7]	HPLC‐FD, confirmed by ELISA	
Açaí (pulp)		—					
Acerola (Barbados cherry)		—					
Banana		0.285	0.009–0.655		[66]	RIA	
					[3]	RIA, HPLC‐MS	
					[67]	HPLC‐FD, ELISA	
					[7]	HPLC‐FD, ELISA	
Guava		—					
Kiwi		0.024			[4]	RIA, HPLC‐FD	
Orange and tangerine		0.150	0.150		[67]	HPLC‐FD, ELISA	
					[7]	HPLC‐FD, ELISA	
Apple and pear		8.356	0.030–67.627		[66]	RIA	
					[4]	RIA, HPLC‐FD	
					[68]	HPLC	
					[69]	RP‐HPLC‐FD	
					[70]	HPLC	
Papaya		0.241			[67]	HPLC‐FD, ELISA	
Mango		0.699			[67]	HPLC‐FD, ELISA	
Watermelon^n^		0.059					
Melon^n^		0.059					
Strawberry and cherry^o^		3.833	0.006–15.000		[66]	RIA	
					[71]	HPLC‐EC	
					[72]	HPLC‐MS	
					[4]	RIA, HPLC‐FD	
					[73]	RIA, HPLC	
					[74]	LLC‐FD, LC–ESI‐MSn	
					[75]	LC‐ESI‐MS/MS	
					[76]	HPLC‐FD	
Peach, plum and nectarine^p^		5.808					
Grape		3.195	0.005–17.500		[77]	HPLC‐FD	
					[78]	HPLC‐FD	
					[79]	HPLC‐MS/MS	
Raisin^q^		3.195					
Tropical fruits (pitanga, mangosteen, soursop, umbu and cupuaçu)		—					
Fruit salad^u^		1.38					
Vegetables							
Pumpkin and squash		0.059			[80]	HPLC‐FD	
Zucchini and chayote^r^		1.166					
Lettuce and Chinese cabbage		0.036			[80]	HPLC‐FD	
Watercress, kale, arugula, spinach and chicory		0.457			[80]	HPLC‐FD	
Cassava, yam and arracacha (cooked)^s^		0.055			[4]	RIA, HPLC‐FD	
Fried cassava^s^		0.055					
Boiled potato		0			[66]	RIA	
					[3]	RIA, HPLC‐MS	
French fries		0					
Beetroot (raw/cooked)		0.002			[3]	RIA, HPLC‐MS	
Eggplant^t^		5.488					
Carrot (raw/cooked)		0.201	0.055–0.494		[66]	RIA	
					[4]	RIA, HPLC‐FD	
Cauliflower and cabbage		0.413	0.107–0.824		[66]	RIA	
					[4]	RIA, HPLC‐FD	
Sweet corn		1.415	1.0–1.878		[66]	RIA	
					[4]	RIA, HPLC‐FD	
Cucumber		1.166	0.025–5.100		[66]	RIA	
					[3]	RIA, HPLC‐MS	
					[4]	RIA, HPLC‐FD	
					[81]	UHPLC‐ESI‐MS/MS	
Bell peppers (red/green)		0.140	0.024–0.51		[82]	LC‐MS/MS	
Green beans^l^		54.79					
Tomato		6.590	0.011–114.5		[3]	RIA, HPLC‐MS	
					[4]	RIA, HPLC‐FD	
					[83]	ELISA, HPLC‐PCD	
					[84]	UHPLC‐Q‐Orbitrap‐MS	
					[85]	UHPLC‐QqQ‐MS/MS	
					[74]	LC‐FD, LC–MS	
					[86]	HPLC‐FD	
Vegetable soup^u^		0.504					
Beverages							
Coffee		69.00	60.0–78.0		[87]	HPLC, LC‐MS‐ESI	
Mate tea and Tereré		—					
Mate tea and black tea		0			[58]	LC–MS/MS	
White tea and green tea		0			[58]	LC–MS/MS	
Fresh fruit juice		0.749	0.185–1.460		[88]	ELISA	
					[78]	HPLC‐FD	
					[70]	HPLC	
Processed fruit juice (canned, boxed and powdered)		0.262					
Processed fruit juice (light/diet)		0.262					
Soda		—					
Diet/light/zero soda		—					
Cachaça (Brazilian sugarcane spirit)		—					
Distilled beverages (vodka, rum and whiskey)		—					
Beer		0.113	0.052–0.333		[89]	ELISA	
					[58]	LC–MS/MS	
					[90]	ELISA, HPLC	
Red Wine		51.270	0.038–423.010		[91]	HPLC‐FD, HPLC‐MS/MS, UHPLC‐ESI‐QTRAP‐MS/MS	
					[92, 93]	UHPLC‐QqQ‐MS/MS	
					[94]	HPLC‐FD	
					[95, 96]	LC‐ESI‐MS/MS	
					[79, 97]	UPLC‐MS/MS, UPLC‐MS/MS (MRM) and Orbitrap HRMS	
Other types of wine		43.292	0.180–390.820		[98]	LC‐ESI‐MS/MS	
					[78]	HPLC‐FD	
					[95, 96, 99]	LC‐MS/MS, LC‐ESI‐MS/MS	
					[97]	UPLC‐MS/MS (MRM) and Orbitrap HRMS	
					[100]	HPLC‐ESI‐MS/MS	
Other foods							
Sugar		—					
Brown sugar/panela (rapadura)		—					
Light sugar		—					
Sweetener		—					
Chocolate (50%–70% cocoa)		0.004			[58]	LC‐MS/MS	
Milk chocolate, bonbon and brigadeiro		—					
Sweets, marshmallow, meringue, candy and taffy		—					
Honey		—					
Popcorn^k^		1.415					
Hot dog and beef/chicken hamburger^u^		1835					
Processed snack (chips‐type snacks)		—					
Pepper (malagueta/finger pepper)		6.688	4.48–7.72		[85]	UHPLC‐QqQ‐MS/MS	
Pudding, ambrosia, dulce de leche, rice pudding and flan^u^		9.41					
Mustard^u^		0.064					
Chocolate drink mix (powdered chocolate)		0.002					
Fried snack (coxinha/rissole/pastel/croquette)^u^		1835					
Large pastry, pot pie and quiche^u^		1835					
Salt		—					
Ice cream		—					
Light ice cream		—					
Canned fruits (fruits in syrup)		0.260					
Guava paste, quince paste, fig paste and peach paste		0.285					
Peanuts, walnuts, Brazil nuts and cashew nuts		3.448					
Fruit jam		3.816					
Soup with rice/pasta^u^		7.951					
Food group	Melatonin (ng/g ou ng/mL)		**Min–max values (ng/g ou ng/ml)**	**Ref.**			**Analytical method**
Dairy							
Whole milk	0.015		0.004–0.039	[51]			ELISA
Skim milk^a^	0.015			[52]			RIA
Semi‐skimmed milk^a^	0.015			[53]			LC‐MS/MS
				[54]			EIA
				[55, 56]			ELISA
				[26]			RIA
Soy milk powder	9.28			[57]			HPLC‐FD
Whole yogurt	0.127			[58]			LC‐MS/MS
Skim/light yogurt^b^	0.127						
Cream cheese^a^	0.015						
Light cream cheese^a^	0.015						
Cheese (mozzarella, provolone, minas, canastra and prato)^b^	0.127						
Cottage cheese^b^	0.127						
Ricotta cheese^b^	0.127						
Meats and Fish							
Mortadella, salami and fatty ham^c^	1.54						
Turkey breast and Chester^d^	1.61						
Beef (steak)	2.1			[59]			HPLC
Beef (cubes and pieces)	2.1						
Chicken with skin	2.3			[59]			HPLC
Skinless chicken	2.3						
Pork	2.5			[59]			HPLC
Lamb, goat meat	1.6			[59]			HPLC
Soy meat and tofu^e^	3.120			[60]			HPLC
Sun‐dried beef (jerked beef)^f^	2.1						
Smoked meats^g^	2.5						
Offal (heart, liver and gizzard)	1.1			[59]			HPLC
Sausage^h^	1.2						
Large sausage and linguiça^h^	1.2						
Boiled chicken egg	6.1			[59]			HPLC
Bacon and pork fat^g^	2.5						
Meatballs	3.18						
Suchi and sachimi	2.42						
Sardines and tuna (canned)^i^	3.7						
Shrimp and shellfish	—						
Salmon	3.7			[59]			HPLC
Codfish^i^	3.7						
Other fish^i^	3.7						
Cereals and Legumes							
French bread	0.342		0.138–0.480	[58]			LC‐MS/MS
				[61]			LC–ESI‐MS/MS
Sliced bread^j^	0.342						
Toast^j^	0.342						
Whole grain bread (rye, wheat and oats)	0.342						
Light bread^j^	0.342						
Sweet bread^j^	0.342						
Cheese bread^u^	0.977						
Breakfast cereal^u^	169.936						
Oats, wheat germ and granola^u^	60.94						
Cereal bar	60.94						
Rice	89.356		11.0–264.0	[62]			HPLC
				[63]			HPLC‐FD
Brown rice	42.95			[63]			HPLC‐FD
Pasta^u^	3.17						
Lasagna, cannelloni and rondelli^u^	2.84						
Gnocchi^u^	3.17						
Polenta and cornmeal mush^u^	42.48						
Fried polenta	42.48						
Canjiquinha (cracked corn porridge)^u^	22.53						
Pizza^u^	2.84						
Cassava flour and breadcrumbs^j^	0.342						
Corn flour^k^	1.415						
Beans and lentils^l^	54.79			[57]			HPLC‐FD
Chickpeas^l^	54.79						
Fats and oils							
Butter	—						
Margarine	—						
Mayonnaise^u^	1.782						
Light margarine and light mayonnaise^u^	0.093						
Olive oil	0.087		0.03–0.119	[64]			LC–MS/MS
				[65]			HPLC‐FD
Canola oil^m^	0.97						
Sunflower oil	0.053		0.050–0.055	[64]			LC–MS/MS
				[65]			HPLC‐FD
Corn oil^m^	0.97						
Soybean oil	0.19			[65]			HPLC‐FD
Pork fat and lard	—						
Fruits							
Avocado	—						
Pineapple	0.311		0.278–0.360	[66]			RIA
				[4]			RIA
				[67]			HPLC‐FD, confirmed by ELISA
				[7]			HPLC‐FD, confirmed by ELISA
Açaí (pulp)	—						
Acerola (Barbados cherry)	—						
Banana	0.285		0.009–0.655	[66]			RIA
				[3]			RIA, HPLC‐MS
				[67]			HPLC‐FD, ELISA
				[7]			HPLC‐FD, ELISA
Guava	—						
Kiwi	0.024			[4]			RIA, HPLC‐FD
Orange and tangerine	0.150		0.150	[67]			HPLC‐FD, ELISA
				[7]			HPLC‐FD, ELISA
Apple and pear	8.356		0.030–67.627	[66]			RIA
				[4]			RIA, HPLC‐FD
				[68]			HPLC
				[69]			RP‐HPLC‐FD
				[70]			HPLC
Papaya	0.241			[67]			HPLC‐FD, ELISA
Mango	0.699			[67]			HPLC‐FD, ELISA
Watermelon^n^	0.059						
Melon^n^	0.059						
Strawberry and cherry^p^	3.833		0.006–15.000	[60]			RIA
				[65]			HPLC‐EC
				[66]			HPLC‐MS
				[4]			RIA, HPLC‐FD
				[67]			RIA, HPLC
				[68]			LLC‐FD, LC–ESI‐MSn
				[69]			LC‐ESI‐MS/MS
				[70]			HPLC‐FD
Peach, plum and nectarine^p^	5.808						
Grape	3.195		0.005–17.500	[71]			HPLC‐FD
				[72]			HPLC‐FD
				[73]			HPLC‐MS/MS
Raisin^q^	3.195						
Tropical fruits (pitanga, mangosteen, soursop, umbu and cupuaçu)	—						
Fruit salad^u^	1.38						
Vegetables							
Pumpkin and squash	0.059			[74]			HPLC‐FD
Zucchini and chayote^r^	1.166						
Lettuce and Chinese cabbage	0.036			[74]			HPLC‐FD
Watercress, kale, arugula, spinach and chicory	0.457			[74]			HPLC‐FD
Cassava, yam and arracacha (cooked)^s^	0.055			[4]			RIA, HPLC‐FD
Fried cassava^s^	0.055						
Boiled potato	0			[60]			RIA
				[3]			RIA, HPLC‐MS
French fries	0						
Beetroot (raw/cooked)	0.002			[3]			RIA, HPLC‐MS
Eggplant^t^	5.488						
Carrot (raw/cooked)	0.201		0.055–0.494	[60]			RIA
				[4]			RIA, HPLC‐FD
Cauliflower and cabbage	0.413		0.107–0.824	[60]			RIA
				[4]			RIA, HPLC‐FD
Sweet corn	1.415		1.0–1.878	[60]			RIA
				[4]			RIA, HPLC‐FD
Cucumber	1.166		0.025–5.100	[60]			RIA
				[3]			RIA, HPLC‐MS
				[4]			RIA, HPLC‐FD
				[75]			UHPLC‐ESI‐MS/MS
Bell peppers (red/green)	0.140		0.024–0.51	[76]			LC‐MS/MS
Green beans^l^	54.79						
Tomato	6.590		0.011–114.5	[3]			RIA, HPLC‐MS
				[4]			RIA, HPLC‐FD
				[77]			ELISA, HPLC‐PCD
				[78]			UHPLC‐Q‐Orbitrap‐MS
				[79]			UHPLC‐QqQ‐MS/MS
				[68]			LC‐FD, LC–MS
				[80]			HPLC‐FD
Vegetable soup^u^	0.504						
Beverages							
Coffee	69.00		60.0–78.0	[81]			HPLC, LC‐MS‐ESI
Mate tea and Tereré	—						
Mate tea and black tea	0			[52]			LC–MS/MS
White tea and green tea	0			[52]			LC–MS/MS
Fresh fruit juice	0.749		0.185–1.460	[82]			ELISA
				[72]			HPLC‐FD
				[64]			HPLC
Processed fruit juice (canned, boxed and powdered)	0.262						
Processed fruit juice (light/diet)	0.262						
Soda	—						
Diet/light/zero soda	—						
Cachaça (Brazilian sugarcane spirit)	—						
Distilled beverages (vodka, rum and whiskey)	—						
Beer	0.113		0.052–0.333	[83]			ELISA
				[52]			LC–MS/MS
				[84]			ELISA, HPLC
Red wine	51.270		0.038–423.010	[85]			HPLC‐FD, HPLC‐MS/MS, UHPLC‐ESI‐QTRAP‐MS/MS
				[86, 87, 88]			UHPLC‐QqQ‐MS/MS
				[89]			HPLC‐FD
				[90, 91]			LC‐ESI‐MS/MS
				[73, 92]			UPLC‐MS/MS, UPLC‐MS/MS (MRM) and Orbitrap HRMS
Other types of wine	43.292		0.180–390.820	[93]			LC‐ESI‐MS/MS
				[72]			HPLC‐FD
				[90, 91, 94]			LC‐MS/MS, LC‐ESI‐MS/MS
				[92]			UPLC‐MS/MS (MRM) and Orbitrap HRMS
				[95]			HPLC‐ESI‐MS/MS
Other foods							
Sugar	—						
Brown sugar/panela (rapadura)	—						
Light sugar	—						
Sweetener	—						
Chocolate (50%–70% cocoa)	0.004			[52]			LC‐MS/MS
Milk chocolate, bonbon and brigadeiro	—						
Sweets, marshmallow, meringue, candy and taffy	—						
Honey	—						
Popcorn^k^	1.415						
Hot dog, beef/chicken hamburger^u^	1835						
Processed snack (chips‐type snacks)	—						
Pepper (malagueta/finger pepper)	6.688		4.48–7.72	[79]			UHPLC‐QqQ‐MS/MS
Pudding, ambrosia, dulce de leche, rice pudding and flan^u^	9.41						
Mustard^u^	0.064						
Chocolate drink mix (powdered chocolate)	0.002						
Fried snack (coxinha/rissole/pastel/croquette)^u^	1835						
Large pastry, pot pie and quiche^u^	1835						
Salt	—						
Ice cream	—						
Light ice cream	—						
Canned fruits (fruits in syrup)	0.260						
Guava paste, quince paste, fig paste and peach paste	0.285						
Peanuts, walnuts, Brazil nuts and cashew nuts	3.448						
Fruit jam	3.816						
Soup with rice/pasta^u^	7.951						

^a^ Used the values for whole milk.

^b^ Used the values for whole yogurt.

^c^ Considered a 70% mixture of pork/beef.

^d^ Considered 70% chicken meat.

^e^ Used soybean values for tofu preparation.

^f^ Same as beef.

^g^ Same as pork.

^h^ Considered a 60% mixture of beef, pork, chicken and offal.

^I^ Values for salmon.

^j^ Same as French bread.

^k^ Used the value for sweet corn.

^l^ Same as red beans.

^m^ Average of all oils.

^n^ Same botanical family as squash.

^o^ Average of strawberries and cherries.

^p^ Same botanical family as apple and cherry, average of apples and cherries.

^q^ Value used for grapes.

^r^ Same botanical family as cucumber.

^s^ Used the value for yam.

^t^ Same botanical family as bell pepper and tomato, average of bell peppers and tomatoes.

^u^ Melatonin content estimated based on ingredient‐level contributions using standard recipes.

Abbreviations: CEC, capillary electrochromatography; EIA, enzyme immunoassay; ELISA, enzyme‐linked immunosorbent assay; HPLC‐EC, high performance liquid chromatography with electrochemical detection; HPLC‐FD, high‐performance liquid chromatography with fluorescence detection; HPLC‐PCD, high‐performance liquid chromatography with postcolumn derivatization; LC/FD, liquid chromatography with fluorescence detection; LC‐ESI‐MS/MS, liquid chromatography–electrospray ionisation–tandem mass spectrometry; LC‐ESI‐MSn, liquid chromatography–electrospray ionisation–mass spectrometry; LC‐MS/MS, liquid chromatography–tandem mass spectrometry; Ref, references; RIA, radioimmunoassay; RP‐HPLC‐FD, reverse‐phase high‐performance liquid chromatography with fluorescence detection; UHPLC‐ESI‐QTRAP‐MS/MS, ultra‐high‐performance liquid chromatography coupled to electrospray ionisation triple quadrupole linear ion trap mass spectrometry; UHPLC‐Q‐Orbitrap‐MS, ultra‐high‐performance liquid chromatography coupled to hybrid quadrupole‐orbitrap mass spectrometry; UHPLC‐QqQ‐MS/MS, ultra‐high‐performance liquid chromatography coupled to triple quadrupole tandem mass spectrometry; UPLC‐MS/MS (MRM) and Orbitrap HRMS, ultra‐performance liquid chromatography coupled to tandem mass spectrometry in multiple reaction monitoring mode, with confirmation by high‐resolution mass spectrometry using orbitrap technology.

Mean dietary melatonin intake was 25554.7 ± 13876.2 ng/day, with a statistically significant difference between men (29215.4 ± 16499.2 ng/day) and women (23810.5 ± 12049.9 ng/day; *p* < 0.001). Coffee, beans/lentils and rice were the main contributors (Table 3). Higher melatonin intake (quintiles) was associated with increased carbohydrate and fibre intake, and reduced intake of protein, fat, cholesterol, monounsaturated fatty acids (MUFA) and saturated fatty acids (SFA) (Table 1).

**Table 3:** Foods with the highest contribution to total dietary melatonin, CUME+ study (2016–2024).

Food/beverage	*R*‐square	Cumulative *R*‐square
Coffee	0.590	0.590
Beans/lentils	0.182	0.772
Rice	0.075	0.847
Beef	0.023	0.870
Pork	0.010	0.880
Red wine	0.008	0.888
Cheese	0.008	0.896
Brown rice	0.007	0.903
Peanuts, walnuts, Brazil nuts and cashew nuts	0.008	0.911
Chickpeas	0.006	0.917

*Note:* Stepwise linear regression.

Regression analyses showed no significant associations between dietary melatonin intake and hypertension, OSA, T2DM or metabolic syndrome. Associations with dyslipidemia and sleep duration were lost after adjustment for sex and age. In contrast, significant associations were observed with obesity and depression (Table 4). These remained robust after adjustment for age and sex (Model 1) and further multivariable adjustments (Model 2). Participants in the second, third and fourth quintiles of dietary melatonin intake had a lower probability of obesity. Additionally, participants with intake between 14,900 and 25,000 ng/day (third and fourth quintiles) were less likely to present depression.

**Table 4:** Associations between quintiles of dietary melatonin intake and health outcomes in the CUME+ study (2016–2024).

Outcomes	Crude model	Model 1	Model 2
Obesity, IRR (95%CI)			
Q1	1 (Ref.)	1 (Ref.)	1 (Ref.)
Q2	0.824 (0.693–0.980)*	0.805 (0.678–0.955)*	0.812 (0.686–0.960)*
Q3	0.743 (0.621–0.888)*	0.692 (0.579–0.827)*	0.720 (0.604–0.857)*
Q4	0.873 (0.737–1.036)	0.801 (0.676–0.950)*	0.795 (0.672–0.941)*
Q5	1.028 (0.874–1.210)	0.909 (0.772–1.070)	0.888 (0.757–1.043)
p‐trend	< 0.001	< 0.001	< 0.001
Obstructive sleep apnoea, OR (95%CI)			
Q1	1 (Ref.)		
Q2	0.907 (0.588–1.399)		
Q3	0.722 (0.455–1.144)		
Q4	0.814 (0.521–1.271)		
Q5	1.001 (0.654–1.527)		
Depression, IRR (95%CI)			
Q1	1 (Ref.)	1 (Ref.)	1 (Ref.)
Q2	0.908 (0.772–1.068)	0.890 (0.758–1.045)	0.902 (0.769–1.059)
Q3	0.805 (0.681–0.953)*	0.782 (0.662–0.925)*	0.787 (0.666–0.930)*
Q4	0.817 (0.691–0.965)*	0.811 (0.686–0.958)*	0.795 (0.673–0.938)*
Q5	0.866 (0.735–1.021)	0.927 (0.785–1.093)	0.875 (0.741–1.033)
p‐trend	0.052	0.003	0.016
Hypertension, IRR (95%CI)			
Q1	1 (Ref.)		
Q2	0.941 (0.774–1.143)		
Q3	0.989 (0.816–1.199)		
Q4	1.188 (0.989–1.426)		
Q5	1.145 (0.952–1.377)		
Type 2 diabetes mellitus, OR (95%CI)			
Q1	1 (Ref.)		
Q2	1.057 (0.724–1.544)		
Q3	0.999 (0.681–1.467)		
Q4	1.001 (0.682–1.468)		
Q5	1.096 (0.752–1.595)		
Metabolic syndrome, OR (95%CI)			
Q1	1 (Ref.)		
Q2	0.933 (0.674–1.291)		
Q3	0.987 (0.716–1.359)		
Q4	1.215 (0.894–1.650)		
Q5	1.147 (0.841–1.564)		
Dyslipidemia, IRR (95%CI)			
Q1	1 (Ref.)	1 (Ref.)	
Q2	1.011 (0.919–1.111)	0.989 (0.901–1.086)	
Q3	1.019 (0.927–1.121)*	0.981 (0.893–1.077)	
Q4	1.051 (0.958–1.155)	1.024 (0.934–1.124)	
Q5	0.900 (0.815–0.994)*	0.910 (0.824–1.004)	
p‐trend	0.017		
Sleep duration, IRR (95%CI)			
Q1	1 (Ref.)	1 (Ref.)	
Q2	0.978 (0.887–1.078)	0.969 (0.880–1.068)	
Q3	0.962 (0.872–1.061)	0.937 (0.850–1.034)	
Q4	0.960 (0.870–1.059)	0.929 (0.842–1.024)	
Q5	1.119 (1.021–1.228)*	1.066 (0.970–1.170)	
p‐trend	0.004		

*Note:* Poisson regression with robust variance and logistic regression. P‐trend indicates a quadratic trend across melatonin intake quintiles.Model 1: adjusted for sex and age. Model 2 adjustments: Obesity: Model 1 + income, binge drinking, physical activity, screen time, sleep duration, weight‐control medications; Depression: Model 1 + income, binge drinking, smoking status, physical activity and sleep duration.

Abbreviations: CI, confidence interval; IRR, incidence rate ratio; OR, odds ratio; Q, quintiles.

* *p* < 0.05. Dietary melatonin (ng/day): Q1: < 14965.4; Q2: 14965.4–20567.7; Q3: 20567.7–26051.8; Q4: 26051.8–34426.5; Q5: ≥ 34426.5.

Given the established role of melatonin in circadian rhythm regulation, we were surprised to observe that individuals in the highest quintile (Q5) initially showed an increased risk of short sleep duration (< 7 h/day) compared with those in the lowest quintile. However, this association was attenuated and lost statistical significance after adjustment for potential confounders. No consistent associations were detected across the other quintiles.

Polynomial contrast analyses revealed significant quadratic associations between dietary melatonin intake and both obesity (*p* < 0.001) and depression (*p* = 0.016) (Table 4), indicating that these relationships may follow a nonlinear pattern across quintiles.

## 4 Discussion

Our findings indicate that dietary melatonin intake may confer protective effects against obesity and depression. Significant quadratic associations were observed, with quintiles Q2–Q4 showing reduced risk of obesity and intermediate quintiles (Q3–Q4) showing reduced risk of depression. These patterns suggest a nonlinear dose–response relationship, supporting the hypothesis that optimal, rather than maximal, melatonin intake may be beneficial for metabolic and mental health.

Similar nonlinear associations have been reported for other nutrients [101, 102, 103, 104, 105, 106]. Moderate intake of methyl donors (riboflavin, folate, choline), carbohydrates, niacin and healthy dietary patterns has been linked to reduced risk of obesity and depression [101, 102, 105, 107, 108]. These findings reinforce the notion that moderation, rather than excess, may be advantageous.

Although dietary melatonin intake was not associated with sleep duration, this null finding may be partly explained by the young age profile of our study population (68.5% aged 18–39 years). Moreover, the lack of data on sleep quality, an important dimension alongside duration, may have limited our ability to detect associations with health outcomes [109, 110]. Taken together, these considerations highlight that the relationship between melatonin intake and health outcomes is complex and context‐dependent, further supporting the notion of nonlinear and multifaceted effects.

Obesity is a multifactorial condition influenced by genetic, environmental and behavioural factors [111, 112, 113]. Individuals with overweight/obesity often consume pro‐inflammatory diets, fewer micronutrients and more ultra‐processed foods [114, 115, 116]. Lifestyle changes remain essential for prevention and treatment [117, 118]. Melatonin may support weight management through antioxidant and anti‐inflammatory properties [119, 120, 121], regulation of energy metabolism [122, 123, 124] and activation of brown adipose tissue [125, 126]. Evidence from animal and clinical studies suggests modest reductions in body weight and adiposity with melatonin supplementation [121, 124, 127, 128].

Dietary melatonin intake in free‐living populations has been estimated in the nanogram range, far below pharmacological doses [14, 15, 27, 28]. Supplementation trials typically use 2–10 mg/day [129, 130, 131], highlighting that mechanisms underlying dietary intake may differ from those of supplementation. Nevertheless, melatonin's high‐affinity receptors, amphiphilic nature and mitochondrial accumulation enable biological effects even at low concentrations [15, 130, 132]. In the gastrointestinal tract, dietary melatonin may influence motility, barrier function and gut–brain signalling [132, 133, 134]. Synergistic interactions with other bioactive compounds (tryptophan, polyphenols, antioxidant vitamins) may further potentiate its effects [131, 135, 136, 137].

**Figure 1:**
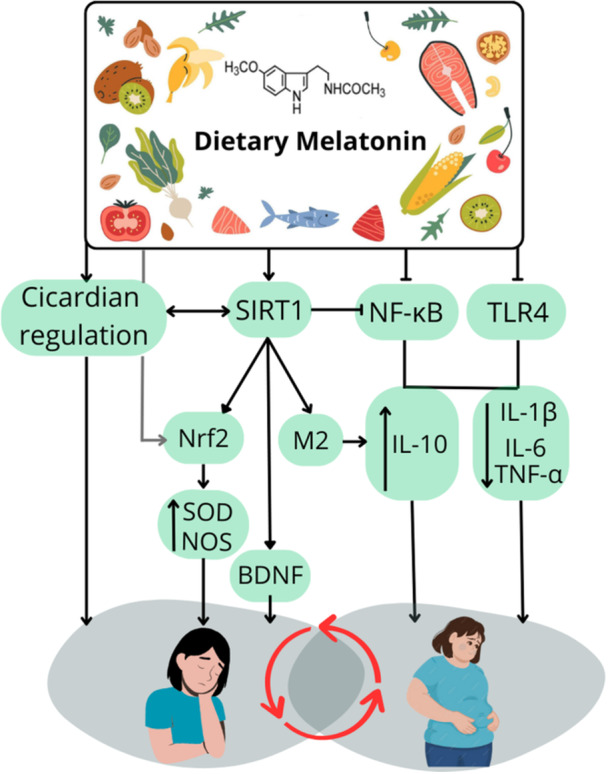
Shared mechanisms linking dietary melatonin to obesity and depression. In addition to its classical role in regulating the circadian cycle, dietary melatonin exhibits antioxidant, anti‐inflammatory and immunomodulatory properties that may benefit conditions such as depression, obesity and obstructive sleep apnoea. The circadian cycle governs various physiological and behavioural processes, including sleep, feeding, hormone secretion, immune function and cellular metabolism. SIRT1 interacts with the circadian clock by regulating core clock genes (such as CLOCK and BMAL1), while its own activity is rhythmically modulated by the circadian cycle through the regulation of NAD biosynthesis—a sirtuin coenzyme and rhythmic metabolite controlled by the circadian clock that plays a key role in linking circadian rhythms to sirtuin activity. Dietary melatonin inhibits NF‐κB activation and modulates TLR4 signalling, reducing proinflammatory cytokines (IL‐6, TNF‐α, IL‐1β) and enhancing IL‐10 expression. It also upregulates Nrf2 and antioxidant enzymes such as nitric oxide synthase and superoxide dismutase, partly through SIRT1‐dependent signalling. Through this pathway, dietary melatonin promotes neuroplasticity by stimulating BDNF expression and favours macrophage polarisation toward the anti‐inflammatory M2 phenotype. Its circadian action influences metabolism, sleep architecture and mood regulation. Figure created by the author based on data from Gasmi et al. [9], Zhuang et al. [153] and Fraiz et al. [154]. Abbreviations: BDNF, brain‐derived neurotrophic factor; BMAL1, Brain and muscle ARNT‐Like 1; CLOCK, circadian locomotor output cycles kaput; IL‐1; interleukin 1 beta; IL‐10; Interleukin 10; IL‐6: interleukin 6; M2, anti‐inflammatory macrophage phenotype; NAD, nicotinamide adenine dinucleotide; NF‐κB, nuclear factor kappa‐light‐chain‐enhancer of activated B cells; NOS, nitric oxide synthase, enzyme responsible for nitric oxide production; Nrf2; nuclear factor erythroid 2–related factor 2; SIRT1, Sirtuin 1; SOD, superoxide dismutase, antioxidant enzyme involved in free radical defence; TLR4, toll‐like receptor 4; TNF‐α; tumour necrosis factor alpha.

Depression is a leading cause of disability worldwide [138, 139, 140], with aetiology involving inflammation, HPA axis dysfunction, neurotransmitter imbalance and gut microbiota alterations [141, 142, 143, 144]. Melatonin may modulate neuroinflammation and circadian regulation, potentially alleviating depressive symptoms [145, 146, 147, 148]. The bidirectional relationship between obesity and depression [149, 150, 151, 152] underscores shared pathways, such as inflammation, neuroendocrine dysregulation and altered sleep, that dietary melatonin may influence. Figure 1 illustrates shared mechanisms linking melatonin to obesity and depression.

Future research should clarify the physiological mechanisms through which dietary melatonin affects metabolic, inflammatory and neurobehavioral pathways. Controlled feeding studies, longitudinal cohorts with biomarker assessments, and mechanistic trials focusing on mitochondrial function, adipose tissue thermogenesis and gut–brain signalling are warranted.

## 5 Strengths and Limitations

This study includes a large, well‐educated sample, with data collected via an online platform. The high educational level likely enhanced questionnaire comprehension and response reliability. Two outcomes with significant associations, obesity and depression, were validated within the cohort [35, 37], strengthening internal consistency. In addition, dietary intake was assessed using a FFQ previously validated for this cohort [34], with precautions to account for seasonal food consumption, reinforcing the robustness of dietary assessment.

Several limitations should be acknowledged. Although dietary melatonin values were assigned to over 80% of FFQ items, data were lacking for some foods. Estimates were derived from published concentrations, which may vary according to analytical methods and agronomic or environmental factors (e.g., soil, irrigation, origin, seasonality, stress exposure) [2, 155]. These sources of variability may affect intake precision. Despite adjustments for multiple confounders, residual bias cannot be excluded.

Health conditions were self‐reported based on prior medical diagnoses, which may have led to underreporting, particularly for conditions that often remain undetected. Nonetheless, outcomes such as depression and metabolic syndrome (including hypertension and obesity) have been validated within the cohort, supporting the reliability of self‐reported data. The relatively young age distribution of participants (68.5% aged 18–39 years) may have limited the detection of associations with age‐related conditions such as hypertension, T2DM and metabolic syndrome, as well as with sleep outcomes. Although analyses on sleep duration were conducted, no significant associations were observed, possibly due to the lack of data on sleep quality, an important dimension alongside duration. Finally, despite the high educational level of the sample, respondent bias cannot be ruled out.

## 6 Conclusion

This study identified inverse associations between dietary melatonin intake and the prevalence of obesity and depression. These findings support hypotheses regarding dietary melatonin's involvement in anti‐inflammatory and metabolic regulation pathways. Nevertheless, longitudinal and experimental studies are required to confirm these associations and clarify underlying mechanisms. Furthermore, the structured compilation of melatonin concentrations in foods provides a valuable resource for future epidemiological research, facilitating intake estimation and hypothesis generation.

## Author Contributions

**Gilmara Alves Zanirate:** study design, data collection, data analysis and interpretation, drafting, editing of the manuscript. **Josefina Bressan:** data analysis and interpretation, editing of the manuscript, project coordination, financial management. **Arieta Carla Gualandi Leal:** data collection, data analysis and interpretation, editing of the manuscript. **Adriano Marçal Pimenta:** data collection and interpretation, editing of the manuscript, project coordination, financial management. **Helen Hermana Miranda Hermsdorff:** study design, supervision, data interpretation, drafting and editing of the manuscript, project coordination, financial management. All authors have approved the final version of the manuscript, agree to be accountable for all aspects of the work, and declare that the content has not been published elsewhere.

## Ethics Statement

The study was approved by the Human Research Ethics Committees of the Federal University of Viçosa (approval number 6.048.479/2023; CAAE 67808923.7.1001.5153).

## Consent

All participants read the informed consent form and provided their agreement online before completing the questionnaire (https://www.projetocume.com.br/questionario).

## Conflicts of Interest

The authors declare no conflicts of interest.

## Supporting information

Supplementary materiallJHND.

## Data Availability

The datasets analysed in the current study are not publicly available, as they are still being used in other studies. However, they are available from the corresponding author upon reasonable request.
