# Supplementary material for: The Content of Dietary Melatonin in 119 Food Items and Its Relationship With Chronic Diseases: Results of the CUME+ Study
Source: J Hum Nutr Diet. 2026 Jan 26;39(1):e70193. doi: 10.1111/jhn.70193 (PMC12835471; doi:10.1111/jhn.70193)
Supplement: Supplementary file 1 — Supplementary materiallJHND. [file JHN-39-0-s001.docx]

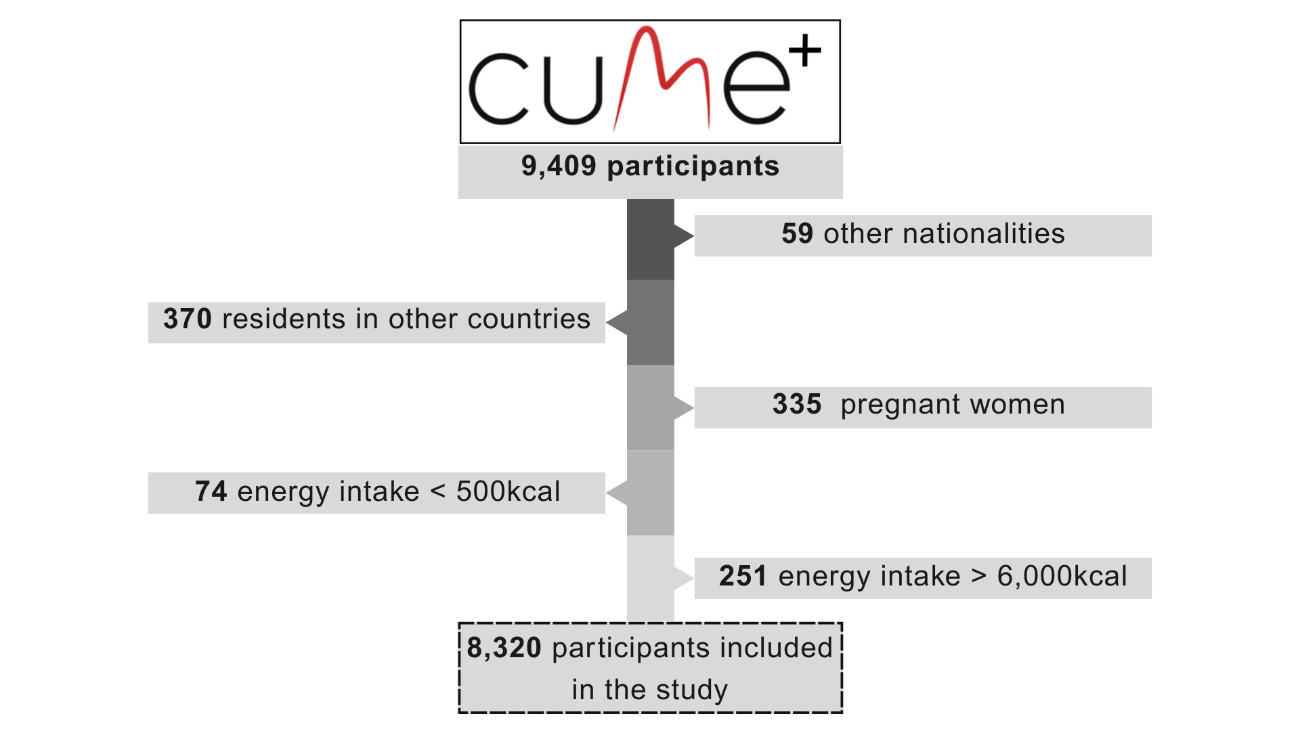


**Supplementary material 1**. Individual selection flowchart for the study participants (2016 – 2024).

**
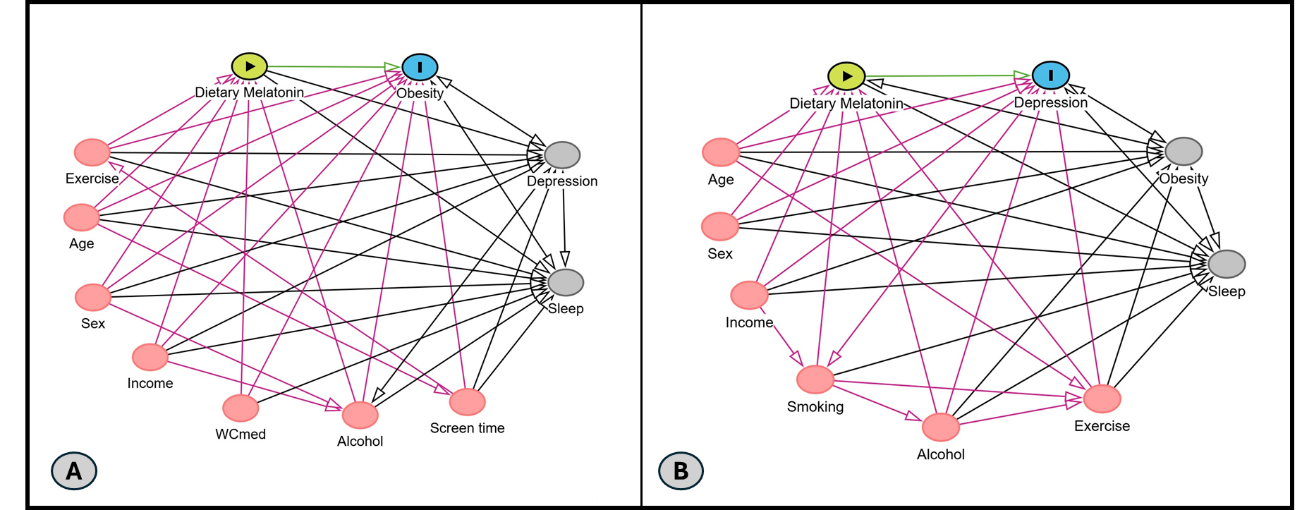
**

**Supplementary material 2**. Theoretical model to summarize the relationships between study variables. A) Obesity; B) Depression. Abbreviations: WCmed, weight control medications. By <https://www.dagitty.net>.


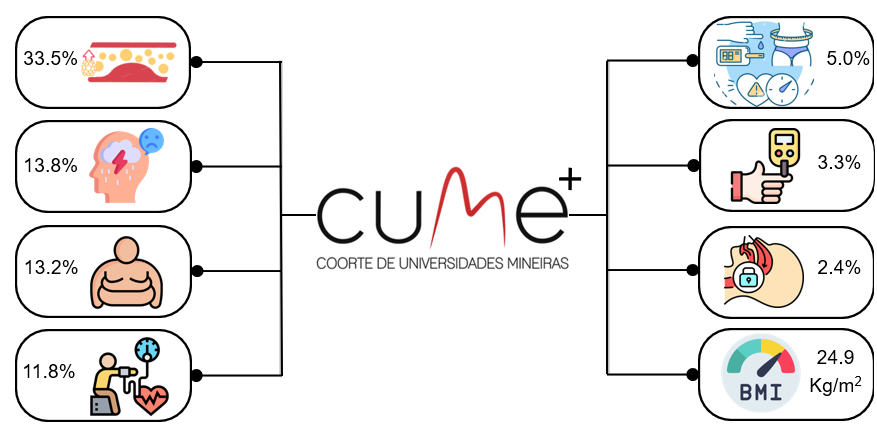


**Supplementary material 3**. Prevalence of chronic health conditions and mean body mass index (BMI) in the CUME+ Study (n = 8,320), 2016–2024. Icons represent dyslipidemia (n = 2,790), depression (n = 1,152), obesity (n = 1,095), hypertension (n = 979), metabolic syndrome (n = 416), type 2 diabetes mellitus (n = 278), obstructive sleep apnea (n = 196), and mean BMI (± 4.7 kg/m²).
